# Ocular hypertension in Axenfeld-Rieger Syndrome

**DOI:** 10.22336/rjo.2020.70

**Published:** 2020

**Authors:** Glenda Espinosa-Barberi, José Francisco Galván González, Alfonso Antón

**Affiliations:** * Institut Català de Retina, Barcelona, Spain; ** Postgraduate and Doctorate School, Universidad de Las Palmas de Gran Canaria, Las Palmas de Gran Canaria, Spain; *** Hospital Universitario de Gran Canaria Doctor Negrín, Department of Ophthalmology, Las Palmas de Gran Canaria, Spain; **** Universitat Internacional de Catalunya, Barcelona, Spain

**Keywords:** Axenfeld Rieger-Syndrome, ocular hypertension, glaucoma, dysgenesis

## Abstract

**Purpose:** to describe a clinical case of ocular hypertension (OHT) in Axenfeld-Rieger Syndrome (ARS).

**Method:** Observational case report of a 43-year-old woman with background of OHT. The data was collected originally with a standardized electronic medical record. A complete ophthalmologic examination was performed.

**Results:** In the biomicroscopy, a posterior embryotoxon, iris atrophy with absence of crypts and irregularity of pigmentation, and discoria in OU were observed. Gonioscopy revealed an open angle with a prominent and anterior displaced Schwalbe line. Ocular fundus (OF) demonstrated small and oblique papillae, with normal neurorretinal ring. Functional tests were normal. The patient did not present systemic pathologies, so the diagnosis of Rieger anomaly was made. The IOP control was achieved with aqueous humor suppressants.

**Conclusions:** Glaucoma is the main cause of visual morbidity in patients with ARS, therefore a complete periodic ophthalmological exam is a priority.

**Abbreviations **:ARS = Axenfeld-Rieger Syndrome, RP = retinitis pigmentosa, IOP = Intraocular Pressure, BCVA = Best Corrected Visual Acuity, OR = right eye, OS = left eye, OU = both eyes, OF = ocular fundus, OCT = optical coherence tomography, VF = visual field, TBC = trabeculectomy

## Introduction

Axenfeld-Rieger syndrome (ARS) constitutes a set of pathologies of embryonic development. In this, structures of the anterior segment are fundamentally affected, occasionally accompanied by other systemic disorders such as dental, craniofacial and umbilical malformations [**1**,**2**].

In 1920, Axenfeld [**3**] described the anterior implantation of Schwalbe line, called posterior embryotoxon, while in 1934, Rieger [**4**] also described iridian and pupillary alterations. 

Incidence is low, affecting 1/ 50.000–100.000 newborns, without ethnic distinction. It presents an autosomal dominant inheritance, affecting PITX2 or FOXC1 gene in 40% of the cases [**5**]. Glaucoma is the most serious ocular complication, appearing in 50% [**6**].

The purpose of this manuscript was to discuss a clinical case and the management of OHT in this pathology.

## Case report

The case of a 43-year-old woman, with OHT treated with timolol 0.5% and bilateral peripheral iridotomy, and family history of open angle glaucoma, was reported.

The best corrected visual acuity (BCVA) was 20/ 20 in both eyes (OU).

**Fig. 1:**
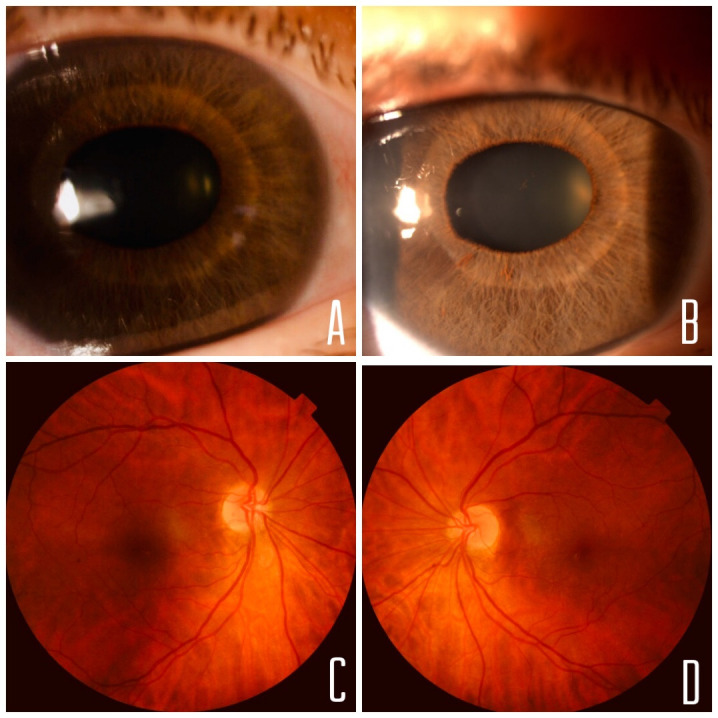
**A,B** Biomicroscopy revealed discoria, iris atrophy, crypt loss and the posterior embryotoxon. **C,D** Color retinographies: oblique and small papillae, with situs inversus. The excavations were normal

In the biomicroscopy, a posterior embryotoxon, a grade 4 anterior chamber (van Herick classification), iris atrophy with no crypts and irregularity of pigmentation, and slight discoria in OU were observed (**Fig. 1 A,B**). The intraocular pressure (IOP) was 33 mmHg in OU. Gonioscopy revealed a grade IV angle according to Shaffer classification, with a prominent and anterior displaced Schwalbe line.

Small, slightly oblique papillae, with vertical excavation of 0.1 and situs inversus in the ocular fundus (OF) were visualized (**Fig. 1 C,D**).

**Fig. 2:**
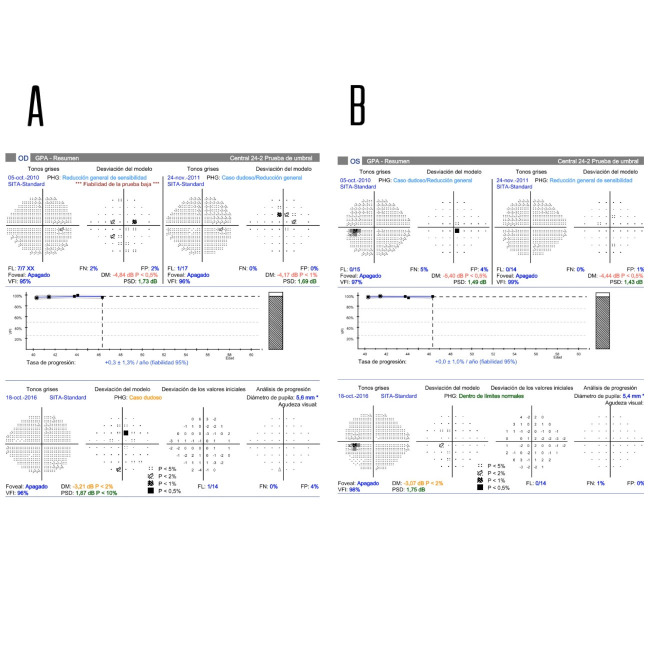
**A,B** Visual field 24.2. Stability of the VFI in the glaucoma progression analysis is observed in both eyes. Doubtful, unconfirmed, and apparently fluctuating central points are seen in the right eye, when compared to previous visual field

Pachymetry values were 584 μm in the right eye (OD) and 572 μm in the left eye (OS). In the visual field 24.2 (VF 24.2), there were no signs of progression compared to previous examinations (OR: VFI 96%, MD -3.21. OS: VFI 98%, MD -3.07) (**Fig. 2**).

Retinal nerve fiber layer optical coherence tomography (OCT) was not assessable due to papillary morphology.

Due to the findings, the patient was diagnosed with ARS. For the treatment of IOP, a fixed combination of timolol 0.5% and brinzolamide 1% was prescribed, remaining at values of 17 mmHg in the OD and 18 mmHg in the OS. 

Subsequently, her daughter has been diagnosed with ARS.

## Discussion

The ARS is a spectrum of three pathologies, whose main common denominator is the anterior segment dysgenesis. These would be Rieger’s anomaly, Axenfeld’s anomaly and Rieger’s syndrome [**7**]. 

In Axenfeld’s anomaly, only embryotoxon and angle disorders were observed. In Rieger’s anomaly, the same Axenfeld alterations were presented, in addition to the affectations at the iris level. In Rieger syndrome, there are also iridocorneal angle and systemic alterations.

Due to the fact that the patient in the case presented had posterior embryotoxon, discoria, alterations in the stroma of the iris and of the iridocorneal angle, we have made the diagnosis of Rieger anomaly.

The cause of this pathology is not yet known. It is believed that it may be due to alterations in the ectoderm development. The posterior embryotoxon is the anterior insertion of the Schwalbe’s line. This can be seen as an opaque line near the corneoscleral limbus [**2**,**8**].

It should be taken into account that the posterior embryotoxon is not always a pathological finding, since it can be found in 15%, without associating other ophthalmological pathologies, but when it is associated with other dysgenesis of the anterior segment, we must think of as the first diagnostic possibility in ARS [**2**,**8**].

This embryotoxon contains collagen covered by spindle-shaped cells. Its origin is from the peripheral iris and reaches the Schwalbe line to insert around it. Other goniodysgenesis that we can find are a rudimentary Schlemm canal, iridocorneal adhesions, etc. [**8**,**9**].

The anomaly and Rieger syndrome are the two pathologies with the highest risk of presenting glaucoma, due to the angular alterations that we can find in them [**7**,**8**].

The inheritance is dominant in most cases, but there are sporadic cases [**1**,**2**]. In our case, it can be said that transmission was dominant, since there was a family history of glaucoma and her daughter was diagnosed with ARS.

This syndrome usually occurs early in life, but patients remain asymptomatic until youth, when the diagnosis of OHT and glaucoma is presented [**9**].

Glaucoma is the main cause of BCVA decrease. Because this pathology affects OU, it is usually bilateral. It presents difficult management, which leads to a reserved visual prognosis [**6**,**8**].

In these cases, the first therapeutic step is made up of the topical suppressors of aqueous humor production, due to angular abnormalities, as we have used in our case [**8**,**9**]. 

In children, treatment is more complicated and is required as initial treatment of the surgery. Goniotomy and trabeculectomy (TBC) require the highest recommendations. Regarding the use of antimetabolites, mitomycin-C combined with TBC has shown better long-term results, making it the technique of choice [**8**,**10**].

## Conclusion

In conclusion, the ARS and the OHT are associated in the case presented. Despite the fact that there are no functional alterations of the optic nerve, it should be considered a pathology at risk of glaucoma as it is a Rieger anomaly. Due to this, periodic complete ophthalmological examinations must be carried out and IOP must be effectively controlled.

**Acknowledgements**

None.

**Sources of Funding**

Authors have not received founding from any organization related (National Institutes of Health (NIH); Wellcome Trust; Howard Hughes Medical Institute (HHMI). 

**Disclosures**

The authors declare that they have no interest in relation to this article.

## References

[1] Berry FB, Lines MA, Oas JM (2006). Functional interactions between FOXC1 and PITX2 underlie the sensitivity to FOXC1 gene dose in Axenfeld-Rieger syndrome and anterior segment dysgenesis. Human Molecular Genetics.

[2] Tümer Z, Bach-Holm D (2009). Axenfeld-Rieger syndrome and spectrum of PITX2 and FOXC1 mutations. European Journal of Human Genetics: EJHG.

[3] Axenfeld TH (1920). Embryotoxon corneaposterius. Klin Monatsbl Augenheilkd.

[4] Rieger H (1935). Dysgenesis mesodermalis corneae et iridis. Z Augenheilkd.

[5] Seifi M, Walter MA (2018). Axenfeld-Rieger syndrome. Clin Genet.

[6] Strungaru MH, Dinu I, Walter MA (2007). Genotype-phenotype correlations in Axenfeld-Rieger malformation and glaucoma patients with FOXC1 and PITX2 mutations. Investigative Ophthalmology and Visual Science.

[7] Dunbar AC, McIntyre GT (2015). Sean Laverick and Brian Steven- son. Axenfeld—Rieger syndrome: a case report. J Orthod.

[8] Rao A, Padhy D, Sarangi S, Das G (2018). Unclassified Axenfeld-Rieger Syndrome: A CASE SERIES and Review of Literature. Semin Ophthalmol.

[9] Kumar P, Senthil S (2019). Progressive High Hypermetropic Shift as a Refractive Surprise Following Glaucoma Filtration Surgery in a Phakic Child With Early-Onset Childhood Glaucoma Associated With Axenfeld-Rieger Anomaly. J Glaucoma.

[10] Mandal AK, Pehere N (2016). Early-onset glaucoma in Axenfeld-Rieger anomaly: long-term surgical results and visual outcome. Eye (Lond).

